# “Mixed connective tissue disease”: a condition in search of an identity

**DOI:** 10.1007/s10238-020-00606-7

**Published:** 2020-03-04

**Authors:** Marta R. Alves, David A. Isenberg

**Affiliations:** 1 Internal Medicine, Department of Medicine, Centro Hospitalar Universitário do Porto, Porto, Portugal; 2 Division of Medicine, Centre for Rheumatology, University College of London, Room 424, 4th Floor, Rayne Building, 5 University Street, London, WC1E 6JF UK

**Keywords:** Mixed connective tissue disease, Anti-U1snRNP, Undifferentiated autoimmune rheumatic disease, Review, Clinical features, Immunologic features, Genetic features, Disease evolution

## Abstract

Mixed connective tissue disease was first described as a new autoimmune rheumatic disease in 1972 based on the claim of a distinct clinical picture associated with anti-RNP antibody positivity. Subsequently, this new entity has divided opinions in the rheumatology community. We have reviewed recent cohort studies with more than 100 patients, comparing the clinical and immunological features, treatment, prognosis and evolution to well-defined autoimmune rheumatic diseases. We also reviewed clinical features of undifferentiated autoimmune rheumatic diseases based on the most recent studies. After gathering and reviewing these data, we discuss whether the designation “mixed connective tissue disease” should be maintained.

## Introduction: a historical perspective

In 1972, Sharp et al. [1] described what was claimed to be a new rheumatological disease. Mixed connective tissue disease (“MCTD”) was reported to be an association of Raynaud’s phenomena, swollen fingers, oesophageal dysfunction, arthralgias, some with non-deforming arthritis, and absent pulmonary and renal disease, in 25 patients with high titres of anti-RNP antibody. This new syndrome consisted of overlapping features between scleroderma, systemic lupus erythematosus, polymyositis/dermatomyositis, with a favourable prognosis, and a low corticosteroid requirement. However, in 1980, Nimelstein et al. [2] reviewed these 25 patients and observed that 8 patients had died, 2 caused by rheumatologic/immunosuppressive-related conditions, and 3 patients could not be found. Most of the remaining 14 patients had evolved into well-defined auto-immune rheumatic diseases (ARD) or an overlap between two ARD. Treatment of these patients was heterogeneous, some requiring high levels of immunosuppression. Levels of anti-RNP antibodies had not in fact been high in all patients when first tested, and these levels did not correlate with disease severity. Several key features of the so-called MCTD were thus challenged casting doubt on the idea that “MCTD” was a distinct disease. Despite the claims made in the initial study, anti-RNP antibodies, even in high titre, lack specificity, the “MCTD” patients often evolve to other well-defined ARD and the idea that low-dose corticosteroid only was required was also challenged [3–9].

Many studies have explored the subject, but no really large-scale prospective studies have been undertaken, and the results of later studies have been just as contradictory [7–9].

Since it was first described, the clinical picture of “MCTD” has changed. Four different classification and diagnostic criteria have been developed (Sharp [10], Alarcon-Segovia [11], Kasukawa [12] and Kahn [13]) and compared in several studies. Which is the most sensitive and/or specific remains controversial [7–9].

Invariably, the discovery of blood test abnormalities, notably antibodies linked to an ARD, has followed its clinical description by many years. Thus, rheumatoid factor was described in the mid-twentieth century, whereas clinical descriptions of rheumatoid arthritis go back to the nineteenth century [14, 15]. Although agreeing that the “MCTD” discovery pathway was unusual and that unanswered questions remain, part of the rheumatological community still considers it a distinct disease [16–19]. This is due to an apparently similar clinical picture in some anti-RNP-positive patients with overlapping features of ARD, a tendency to the insidious development of PAH (pulmonary arterial hypertension) and ILD (interstitial lung disease), and relatively uncommon renal or neurological involvement. A genetic association between HLA haplotype and anti-U1RNP antibodies was discovered, which some have interpreted as supporting the concept of MCTD [20–22]. However, other studies demonstrated that this linkage did not correlate with clinical disease expression, merely with antibody production [23, 24].

## Data review

We have reviewed cohort studies with a significant number of patients (*n* > 100) and a follow-up period time of more than 5 years in an attempt to draw more tangible conclusions. Four studies were identified and compared. Studies with more than 100 patients are uncommon, though a study that analyses anti-RNP positive patients, with 50 MCTD patients, was also included. Cohort studies of Gunnarson et al. were not reviewed as they only address pulmonary disease in “MCTD”.

Cappelli et al. [25] identified 161 patients in 15 tertiary Italian centres. Patients included had “MCTD” diagnosed according to expert opinion and upon chart review were classified in accordance with the three main set of criteria (Kasukawa, Alarcón-Segovia and Sharp). Sixteen patients (9.9%) did not fulfil any diagnostic criteria after chart review. Every patient was subsequently evaluated with a mean disease duration of 7.9 ± 5.9 years (range 1–31 years). Patients that fulfilled diagnostic criteria for a well-established ARD at initial diagnosis were excluded. Notably, patients fulfilling criteria for “MCTD” and another ARD at study end (2008) were considered to have “MCTD”. Therefore, overlap and evolution to other ARDs were only considered to have occurred in patients no longer satisfying any “MCTD” criteria. In 22 patients (14%), no anti-RNP data were provided.

In the Reiseter et al. [26] cohort study, 147 patients were identified from the Norwegian nationwide “MCTD” database, and 118 were studied (13 patients were lost and 16 deceased before revaluation). Inclusion criteria were age above 18 years, fulfilment of at least one of the three sets of criteria for “MCTD” (modified Sharp’s criteria, Alarcón-Segovia, Kasukawa) and exclusion of another ARD. Patients submitted to a protocol evaluation when the database was created (*t*1) and revaluated for the study (*t*2). At *t*1, the mean disease duration was 10(± 8), and at *t*2, it was 17 (± 9) years. More than 90% of the 118 patients had disease duration > 8 years and were observed for > 5 years. Disease conversion was defined as change of antibody profile together with concordant clinical features of another ARD. In those patients in whom more than one specific autoantibody was identified, the dominant antibody specificity was determined together with the clinical features. It is not clear whether “MCTD” classified patients also fulfilled criteria for other ARD. Remission was considered and was evaluated using a combination of SLE and SSc validated activity criteria, though neither is validated for use in “MCTD”. Remission was defined as a Systemic Lupus Erythematosus Disease Activity Index 2000 (SLEDAI-2K) equal to zero and a European Scleroderma Trials and Research group score (EUSTAR) inferior to 2.5. The main goal of this study was to compare the characteristics at t1 of “MCTD” stable patients and disease converters.

Hajas et al. [27] recruited 280 patients followed at the Division of Clinical Immunology, University of Debrecen—Hungary, between 1979 and 2011. Mean follow-up time was 13.1 (± 7.5) years, and the Alarcón-Segovia criteria set was used for inclusion. It is not clear whether patients fulfilling other ARD criteria were excluded, nor which criteria were used to evaluate disease conversion. Disease activity was assessed using the Systemic Lupus Activity Measure index (SLAM), which is not validated for use in “MCTD”.

Ungprasert et al. [4] used the resources of the Rochester Epidemiology project to study the anti-U1RNP positive antibody Olmsted County, Minnesota, population. Between 1985 and 2014, 264 patients were identified. Inclusion criteria were age above 18 years and a positive anti-RNP antibody test. “MCTD” diagnosis was appraised using and comparing all four known criteria, without fulfilling other ARD criteria. Fifty patients fulfilled at least one of the “MCTD” criteria. Disease duration was characterized using median and interquartile range: 10 years (4.9; 14.3). The criteria used to evaluate disease conversion were not explained. Disease activity was not assessed.

Szodoray et al. [28] evaluated 201 patients from a single centre. Fulfilment of Alarcón-Segovia criteria was required for individuals in the study, and the mean follow-up time was 12.5(± 7.2) years. Although this was a longitudinal study, it is not clear whether clinical features and antibody profile described were at diagnosis or after follow-up. Patients were divided into three pathologic groups, with statistically significant different incidence of organ damage. Evolution to other ARD was not considered although Sjogrën’s syndrome was described in 36.8% of patients. Treatment was not evaluated. Owing to the lack of clarity about the clinical data timing and the absence of evaluation of evolution to other ARDs, this study is not further discussed in this review.

Frandsen et al. [29] evaluated 151 patients with an anti-RNP positive antibody. In this study, anti-RNP antibody testing was undertaken by passive hemagglutination, which differs from all other studies which used ELISA. Furthermore, the “MCTD” diagnostic criteria used was that proposed by Rasmussen et al. and differs from all the other studies. Thus, we have not considered this study either in our analysis.

### Clinical features

Table 1 summarizes and shows comparison of the clinical features described at diagnosis and the cumulative frequency after follow-up time in each study. Mean follow-up time is provided at each evaluation.

**Table 1:** Clinical features described in each study

	Cappelli et al. (25)		Reiseter et al. (26).		Hajas et al. (27)		Ungprasert, et al. (4)	
Clinical features	At diagnoses	2008	*t*1	*t*2	At diagnoses	2011	At diagnoses	At 10 years
*n*	161		118 (134–16 deceased)		280		264 (50)^a^	
Male gender	9%		24%		7.5%		16%	
Age, years, mean (SD)			44 (14)		53.1 (12.6)		48.1 (15.7)	
Follow-up time, years, mean (SD)	7.9 (5.9)		10 (8)	17 (9)	13.1 (7.5)		8.3 (3.4; 14.1)^b^	
Raynaud’s phenomenon	93.2%	85.1%	99%		50,3%	57,5%	80%	
Arthritis	73.9%^c^	49.7%	78%		65.3%^d^	89.6%^d^	86%	86.0%
Puffy hands	72.7%	46.0%	92%		53.6%	55.6%	64%	83.3%
Sclerodactyly	29.2%	43.0%	28%		35.3%	41.8%	14%	26.8%
Hypomotility or dilatation of oesophagus	34.8%	45.3%			38.9%	49.6%		
Interstitial lung disease	28.6%	44.1%	34%		0.7%	47.1%	18%	27.8%
Pulmonary arterial hypertension					0.0%	17.8%	2%	6.9%
Pleuritis	21.7%	18.6%	12%		13.9%	29.6%	6%	14.9%
Pericarditis			9%					
Facial erythema	19.9%	16.8%	44%		32.9%^e^	36.4%^e^	4%	4.0%
Lymphadenopathy	18.0%	13.7%					4%	6.1%
Neurological involvement	5.6%	11.2%			12.5%	20.0%	0%	4.1%
Myositis	27.9%^f^	19.2%	30%		13.5%	32.5%	24%	30.6%
Renal involvement (nephritis)	6.8%	9.9%			0.0%	3.9%	0%	6.0%
Anti/phospholipid syndrome	NR	NR	NR	NR	3.9%	25.7%	NR	NR
Cardiovascular involvement	NR	NR	NR	NR	7.5%	35.0%	NR	NR
Cancer	NR	NR	NR	NR	0.0%	16.0%	NR	NR
Leukopenia	24.8%	26.7%	31%				44%	5.8%
Thrombocytopenia			14%		6.7%	22.5%	0	62.5%

*NR* not reported

^a^264 anti-RNP-positive patients studied, 50 where diagnosed with MCTD

^b^Interquartile range

^c^Arthralgia was also considered

^d^Erosive and non-erosive arthritis

^e^Considered also photosensitivity, telangiectasia and hyper-pigmentation

^f^Only elevated CK was considered

Comparing the most striking features of patients with “MCTD”, at diagnosis and cumulative frequency after around 10 years of follow-up, it is noticeable that they often differ substantially across the various studies. Puffy hands vary between 53 and 72% at presentation and 46% and 92% after follow-up. Raynaud’s phenomenon was present in between 50.3 and 93.2% at presentation and 57.5% to 99% after follow-up. Arthritis at presentation occurred between 65.3 and 86%, 49.7 and 89.6% after follow-up. Oesophageal hypomobility or dilation occurred in 34.8% to 38.9% at presentation and 45.3 to 49.6% during follow-up. Evolution to ILD and/or PHA is reported in some, but not all, studies. Some believe it to be a characteristic of patients with “MCTD” [30–32], but clearly in Table 1, after 10 years follow-up, this evolution is also not homogenous. PHA was only evaluated in two studies and occurred in between 6.9 and 17.8% of patients. ILD is addressed in 4 of the 5 studies and occurred in 27.8% to 47%.

### Immunologic and genetic features

It is generally assumed that a positive anti-U1RNP antibody is necessary for a diagnosis of “MCTD” to be made. However, it is not exclusive to this condition. In our own group of Systemic Lupus Erythematosus (SLE) patients, anti-RNP antibodies were present in 35% [33]. Ungprasert et al. studied 264 anti-RNP-positive patients, and a majority had other ARD, and only 18.9% fulfilled at least one of the “MCTD” criteria. The non-“MCTD” patients had other rheumatologic diseases (SLE 58%; RA 25%; UARD 15%; Sjogrën 7%; primary Raynaud’s 7%; cutaneous lupus 6%; systemic sclerosis syndrome 2%) or, importantly, lacked any overt rheumatologic symptoms. It is also important to highlight that disease conversion (reviewed below) may also occur in anti-RNP-positive patients.

Several smaller studies have reported that “MCTD” patients have a high anti-RNP titre [1, 16, 19, 32]. However, Reiseter et al. compared the characteristics between “MCTD” stable patients and converters, in 147 patients, and reported higher median anti-RNP titles in disease converters—median (IQR): 27(5–66) in “MCTD” stable; 104(25–240) in disease converters. Furthermore, Ungprasert et al. also described high titres of anti-RNP antibodies in 46% of their 264 anti-RNP-positive population, but only 29% of the high anti-RNP pool (35/121) had “MCTD”.

Other antibodies (anti-dsDNA, Sm, Scl70, SSA/Ro, SSB/La, CCP, cardiolipin, β2GP, endothelial cell antibodies) have been reported in patients with MCTD with variable frequencies. Several studies have attempted to link different antibody profiles to clinical features and evolution to well-defined ARD [26, 28]. However, this is not the focus of this review and will not be further discussed here.

A cornerstone in the advocacy of the existence of “MCTD” is the association between antibody production and HLA haplotypes. Different studies have demonstrated an association between the presence of anti-U1RNP antibody and an HLA-DR4-specific haplotype [20–22, 34]. Genth et al. [23] studied 35 patients with anti-U1RNP-positive ARD, with a mean disease duration of 6.3 years. In this study, HLA-DR4 was not associated with disease expression as it did not differ between patients classified as “MCTD” or other ARD. Gendi et al. [24] evaluated 39 “MCTD” patients after a 10-year follow-up time and found that 25 of those patients had evolved to another ARD, and that HLA DR4 was present in 10 of those 25 converters and 9 of the 14 non-converters. They also found an association between HLA haplotype and evolution towards a well-established ARD. A more recent study [34] evaluated 155 patients with a mean duration 11.6 years (SD 8.4) with the diagnosis of “MCTD”, comparing to 282 healthy controls, 96 SLE, 95 SSc and 84 PM patients. The presence of anti-U1RNP antibody in non-“MCTD” patients was not reported. HLA-DR4 was associated with “MCTD” (OR 2.8), unlike SLE that is described as being negatively associated with this HLA haplotype. HLA-B alleles were also evaluated and HLA-B8 was positively associated with “MCTD” (OR 2.0) SLE (OR:2.4) and PM (OR3.3).

### Evolution to well-established autoimmune rheumatic diseases

Disease stability is a core argument in favour of the existence of “MCTD” as an independent ARD. The aforementioned studies address this issue but report conflicting results (Table 2). Cappelli et al. noted a diagnostic conversion frequency of 42.1%, but this value was calculated considering that the “MCTD-stable” patients encompass those who also fulfil well-established ARD criteria. If the “MCTD-alone” patients are considered, the conversion frequency is 85.6% in this follow-up study over 7.9(± 5.9) years.

**Table 2:** General characteristics of the evaluated studies

Name of study	*n*	Follow-up time—mean (SD)	Classification criteria at diagnosis				Treatment	Outcome		
			Sharp [10]	Kasukawa [12]	Alarcón-Segovia [11] (%)	Kahn [13]		“MCTD” stable	Converters	Deceased and other observations (*n*, %)
Cappelli et al. [25]	161	7.9 (5.9)	41.6%	75.2%	73.3	Not used	58%^a^ immunosuppressant 82%^b^ steroids 45% antimalarial drug	57.9%^c^ (14.4% MCTD alone; 40.5% MCTD + other ARD)	42.1%	Deaths in the MCTD group (*n* = 161) 5/161 = 3.1%
Reiseter et al. [26]	118	17 (9)	96.6%	85.6%	91.6	Not used	Not reported	88.1%	11.9%	Loss of follow = 13 Deaths in the MCTD group (*n* = 134) 16/134 = 11.9%
Hajas et al. [27]	280	13.1 (7.5)	Not used	Not used	100	Not used	78.2% high-dose steroid^d^ 74,6% cytotoxic agent^e^ 15% anti-TNF	100%	0	Deaths in the MCTD group (*n* = 280) 22/280 = 7.9%
Ungprasert et al. [4]^f^	264 (50)^g^	8.3 (3.4; 14.1)^h^	28%	72%	72	54%	Not reported	90%	10%	Deaths in the MCTD group (*n* = 50) 5/50 = 10%

^a^Cyclophosphamide, cyclosporine, mofetil mycophenolate, azathioprine, methotrexate and leflunomide

^b^Dose not explicit

^c^Percentages according to Kasukawa criteria set as it was considered the most accurate in this study

^d^≥ 1 mg/kg/day methylprednisolone

^e^Methotrexate, cyclophosphamide, azathioprine

^f^Cohort study of anti-RNP-positive patients

^g^264 RNP positive. 50 fulfilled MCTD criteria at follow-up initiation

^h^Median; interquartile range

In the Reiseter et al. study, the conversion frequency was 11.9% after a follow-up of 17 ± 9 years. As above, considering that conversion definition is not strict, it is not clear whether “MCTD-stable” patients also fulfilled other ARD criteria. It also considered remission on treatment (prednisolone ≤ 5 mg/day; azathioprine, methotrexate, mycophenolate mofetil) or not (treatment hydroxychloroquine, calcium channel blockers, intermittent non-steroid anti-inflammatories were allowed). The absence of the anti-RNP antibody was considered a remission marker. Therefore, patients who once fitted the criteria for “MCTD” but no longer did, due to absent disease activity, were considered to have “MCTD”, regardless of their anti-RNP antibody status. Furthermore, the most common symptoms in patients with active “MCTD” disease were arthritis, rash and alopecia. Conversion to SLE occurred in 5 of 118 patients (4.3%) that developed high anti-dsDNA titres and low complement. RA was diagnosed in 4 of 118 patients (3.4%) with positive anti-CCP and bone erosions on X-ray, and they concluded that bone erosions are not common in “MCTD” and should raise suspicion about differentiation to other ARD. These results raise several questions, namely whether: anti-RNP antibodies were an intermittent finding and had no relation to therapy; therapy was concealing clinical manifestations that would otherwise occur and enable evolution into a well-defined ARD; if the “MCTD-stable” patients fulfilled other ARD criteria and, in fact, had an well-defined ARD.

Hajas et al. described an “no disease conversion” over a period of 13.1(± 7.5) years. It is not clear which criteria were used to evaluate disease conversion. As described below, 78.2% patients were treated with high-dose steroids, 74.6% with cytotoxic agents and 15% with anti-TNF agents. In this cohort, 11 patients had renal disease with a biopsy showing thrombocytopenic thrombotic purpura/haemolytic-uremic syndrome (TTP/HUS) in 3 patients, ISN class II (5 patients) or class V glomerulonephritis (3 patients). Anti-dsDNA antibodies were present in 3.2% and anti-Sm antibodies in 6.7%, but complement levels were not measured. An association with anti-phospholipid syndrome (APS) was also described in 72 of 280 patients (25.7%), in contrast to previous reports (19,35) that described patients with positive APS antibodies without clinical APS criteria. In contrast to Reiseter et al., erosive arthritis was diagnosed in 17.5% of patients, after 5 to 10 years of the “MCTD” diagnosis. An anti-CCP antibody was positive in 18.9% at the end of the follow-up. Analysing this study, although the data are scattered, it is debatable whether some of these “MCTD” patients would not have fulfilled other ARD criteria.

Ungprasert et al. reported a 10% conversion rates, but again no description of the criteria used to determine whether other ARD had developed, nor which treatment was given.

### Treatment and prognosis

The treatment of “MCTD” is relatively little discussed. In Hajas et al., 219 of 280 (78.2%) patients were treated with high-dose steroid (≥ 1 mg/kg/day methylprednisolone), 209 (74.6%) with cytotoxic agent (methotrexate, cyclophosphamide, azathioprine) and 42 (15%) with anti-TNF. Cappelli et al. reported 58% patients treated with immunosuppressants (cyclophosphamide, cyclosporine, mofetil mycophenolate, azathioprine, methotrexate, leflunomide), 82% requiring glucocorticoids (no dose reported) and 45% of patients treated with antimalarial drugs. Thus, in these studies, most patients needed immunosuppressive treatment. Treatment varied with organ evolvement, in accordance with current well-defined ARD guidelines.

Ungprasert et al. reported 10% deaths, but the overall mortality rate was not different from the general population. Reiseter et al. also reported 11.9% deaths in their group, although these patients had an older mean age and higher prevalence of pericarditis and ILD. Hajas et al. described 7.9% deaths in their study. The major cause of death was PAH, followed by cardiovascular events and TTP/HUS. Overall, the prognosis appears to be connected to the presence of pulmonary disease.

## Diagnostic criteria and current guidelines

Overall, it is hard to escape the conclusion that there is no current evidence or agreement about the optimal criteria for diagnosis, follow-up or treatment strategies. It may be argued that larger studies are needed to allow these conclusions. It is evident that these patients might require careful periodic lung involvement evaluation.

Hajas et al. used one set of diagnostic criteria, whereas all the other studies used several different diagnostic criteria sets. No agreement on which one is the most sensitive criteria set was achieved, when comparing the studies analysed.

## Undifferentiated autoimmune rheumatic disease overview

UARD is a term used to capture those patients with clinical features and antibodies compatible with an ARD, but who do not fulfil the criteria for any well-defined individual ARD. Currently, there are no globally accepted diagnostic criteria [35, 36].

Symptom frequency described in several cohort studies are scattered, as in “MCTD”, although most studies have 5-year follow-up time or less [36]. Arthritis frequency ranges from 15.2 to 33%, Raynaud’s phenomenon from 6.3 to 58.8%, malar rash 3% to 25.3% and photosensitivity 17 to 40.5% [36]. Pulmonary disease is not described as frequently as in the “MCTD” cohorts, but UARD cohorts have a shorter follow-up time, some of them are considerably larger, and pulmonary disease may not have been systematically sought. In contrast, Kinder et al. evaluated 280 patients with ILD, 53 (18.9%) of those patients had a well-defined ARD associated (only 1 patient had “MCTD”—0.4%) and 28 (10%) patients had UARD [37]. Similarly, Alhamad et al. reported a comparison of patients with idiopathic pulmonary fibrosis versus 67 patients with ARD-related ILD (ILD-ARD). Among these ILD-ARD patients, 33% were described as UARD and only 6% as “MCTD”. Thus, pulmonary disease in UARD may have a bigger incidence than that described in UARD cohorts. More studies are needed. Oesophageal hypomobility is rarely reported [38], and sometimes, only dysphagia is mentioned [39], with a range from 1 to 7.2%. Iudici et al. addressed the quality of life in 46 patients with UARD (follow-up 7.5 ± 5.5 years), among whom, gastrointestinal symptoms (dyaphagia/heartburn and early satiety) were reported in 32,6% [40].

Anti-RNP antibodies are reported in the UCTD cohorts, with no consistent clinical significance [38, 39, 41, 42]. Evolution to well-established ARD ranged between 5 and 68%. Some studies describe a relation between evolution to well-established ARD with antibody association at presentation or new antibody appearance during follow-up [38, 39, 42].

As with “MCTD”, patients are treated similarly to those with the well-established ARD [38, 39, 41, 42].

## Final discussion

Following the original claims about the existence of a new entity, “MCTD” rapidly became very popular. Perhaps this was because rheumatologists sought to “accentuate the positive”, when explaining to their patients that while they had a disease of their immune system, its symptoms were relatively mild, the corticosteroid requirement was low, the symptoms annoying but not serious and, most importantly, prognosis was good. Sadly, it is very clear, from this and earlier reviews [7–9], that none of these beliefs really stands up to critical scrutiny and nor does the implication that high levels of antibodies to RNP must be present to fit any diagnostic criteria. The experience that we and many other groups have had is that there are many individuals with high levels of antibodies who do not have the “classical” symptomatology of the so-called MCTD, and likewise, there are patients with these symptoms who have either low titres or no evidence of anti-RNP antibodies. The original claim that there were no pulmonary or renal disease features in these patients is clearly incorrect, especially with regard to the former, and it is notable that pulmonary hypertension seems to be a relatively common cause of death in patients still diagnosed with “MCTD”. The requirement for corticosteroid, and other immunosuppressive drugs, which were said to be low/modest is also untrue.

Although no direct comparison has been made, reviewing the “MCTD” and UARD literature, there are no striking differences regarding clinical presentation, evolution, treatment, and prognosis. It would be interesting to compare all the undifferentiated patients (“MCTD” and UARD) anti-RNP positive and negative.

## Conclusion

While we do not pretend that there are no patients who may have high levels of antibodies to RNP and whose clinical features fall within the “MCTD” description, many of these patients, as clearly indicated by long-term follow-up studies, “evolve” into other more specific ARDs. We take the view that the patients, whose clinical features remain stable, would best be described as having an undifferentiated ARD. The term MCTD seems to us thoroughly discredited and does not better define these patients.

Further and larger studies are still needed to assess the significance of anti-U1RNP antibodies in ARD. Further long-term cohort studies encompassing all undifferentiated patients are also needed to ascertain diagnostic criteria, optimal follow-up, treatment and prognosis.
